# Obesity in American Indian and Mexican American Men and Women: Associations with Blood Pressure and Cardiovascular Autonomic Control

**DOI:** 10.1155/2013/680687

**Published:** 2013-08-19

**Authors:** José R. Criado, David A. Gilder, Mary A. Kalafut, Cindy L. Ehlers

**Affiliations:** ^1^Department of Molecular & Cellular Neuroscience, The Scripps Research Institute, 10550 North Torrey Pines Road, TPC-32, La Jolla, CA 92037, USA; ^2^Stroke Center and Division of Neurology, Green Hospital, Scripps Clinic, La Jolla, CA 92037, USA; ^3^Department of Molecular & Experimental Medicine, The Scripps Research Institute, La Jolla, CA 92037, USA

## Abstract

Obesity is a serious public health problem, especially in some minority communities, and it has been associated with an increased risk of cardiovascular diseases. While obesity is a serious health concern in both American Indian and Mexican American populations, the relationship between obesity and cardiac autonomic control in these two populations is not well understood. The present study in a selected sample of American Indians and Mexican Americans assessed associations between obesity, blood pressure (BP), and cardiovascular autonomic control. Cardiovascular autonomic control, systolic and diastolic mean BP, and body mass index were obtained from one hundred thirty-two American Indian and Mexican American men and women who are literate in English and are residing legally in San Diego County. Men had a significant greater systolic and diastolic BP and were more likely to develop systolic prehypertension and hypertension than women. Obese participants showed greater mean heart rate (HR) and systolic and diastolic BP than nonobese participants. Obese men also exhibited greater cardiac sympathetic activity and lower cardiovagal control than obese women. These results suggest that obesity and gender differences in cardiovascular autonomic control may contribute to risk for cardiovascular disorders in this sample of American Indians and Mexican Americans.

## 1. Introduction

The prevalence of obesity in American Indians is high and is generally associated with insulin resistance and diabetes [1–3], but generally only in the past few generations [4]. This increase in the incidence of obesity in American Indians may be due in part to the relative abundance of high fat, high calorie food, and a shift from an active to a more sedentary lifestyle [2]. There is also evidence of greater odds of obesity among US-born Mexican Americans, in comparison to first generation Mexican Americans [5]. The National Health and Nutrition Examination Survey (1999–2002) reported a 40% prevalence of overweight in Mexican-origin children between the ages of 6 and 19 (versus 28% for non-Hispanic whites) [6]. This study also found a 22% prevalence of obesity in Mexican Americans (versus 14% for non-Hispanic whites). These findings suggest that obesity is a serious health concern in both American Indian and Mexican American groups.

Obesity has been associated with an increased risk of cardiovascular diseases including hypertension, arrhythmias, coronary artery disease, and stroke [7]. American Indians and Hispanics, together with African Americans, have higher stroke risks and stroke occurrence at an earlier age, than non-Hispanic whites (see [8]). Moreover, both American Indians and Mexican Americans have shown an increase in risk factors for cardiovascular disease such as alcohol and tobacco use [9]. The synergistic effects of obesity and alcohol/drug abuse on the development of cardiovascular disease are not well understood. Understanding the relationship among these risk factors may provide important insight into the prevention and treatment of these conditions.

Evaluation of noninvasive cardiovascular autonomic control based on measures of heart rate variability (HRV) is playing an important role elucidating the role of the autonomic nervous system (ANS) in several neurological, cardiovascular, and psychiatric disorders (see [10–12]). HRV, the beat-to-beat variation in heart rate, is the result of the dynamic interaction between the sympathetic (SNS) and parasympathetic (PNS) divisions of the ANS at the Sino-Atrial (SA) node of the heart [11, 13]. There is evidence to suggest that the ANS may play an important role in blood pressure (BP) regulation and during the early stages of hypertension [14–16]. Studies have shown that reductions in overall HRV amplitude and measures of cardiovagal control may precede the development of hypertension and increase the risk of cardiovascular disease (see [12]). A reduction in overall HRV amplitude is also considered to be an independent predictor of mortality in patients with postmyocardial infarction [17]. However, the contribution of the ANS to risks of cardiovascular disease is not well understood.

Time- and frequency-domain metrics of HRV have been used to assess cardiovascular autonomic control in psychophysiological studies (see [11, 12]). While some of these measures provide estimates of overall variability (e.g., standard deviation of the normal-to-normal (SDNN) heart beat), metrics that index respiratory-linked changes have been developed to provide a more accurate estimate of cardiac vagal control. For instance, vagal influence of respiratory-mediated HRV, also known as respiratory sinus arrhythmia (RSA), is an estimate of vagal efferent activity to the SA node of the heart. Several time-domain metrics of HRV that index respiratory-linked cardiovagal control include the percentage of the absolute differences between consecutive IBIs that are greater than 50 ms (pNN50; [18]); the square root of the mean squared successive differences between IBIs (RMSSD; [19]); and RSA, defined as the natural log of band-limited (0.12–0.40 Hz) variance of IBI time series [11]. In the frequency domain, it has been demonstrated that high-frequency (HF) HRV (HF-HRV) in the respiratory frequency band provides an index of cardiovagal control (see [20, 21]). In contrast to the cholinergic-mediated effects of the vagus nerve, the effects of the SNS on the SA node are diffuse and slow [22]. Toichi and colleagues [23] have developed a method to assess the sympathetic influence over HRV by estimating the cardiac sympathetic index (CSI). Studies have shown that beta-adrenergic antagonism with propanolol significantly reduced the CSI in individuals in standing and sitting conditions [23]. In addition, the low-frequency (LF) HRV (LF-HRV) is a frequency-domain method that indexes a combination of cardiosympathetic and cardiovagal controls (see [20, 21]). While these psychophysiological research methods provide estimates of cardiac vagal and sympathetic control, clinical assessment of cardiovascular autonomic control has been also used to index cardiovascular PNS control [10, 24, 25]. One of the most commonly used methods, the HR response to deep breathing (HR_DB_), requires participants to breathe at a rate of 6 breaths per minute (bpm) over a short interval (<90 sec). The estimate of cardiovagal control is based on the mean HR range. These clinical methods have been used to assess cardiovascular autonomic control in patients with autonomic disorders [10, 24, 25]. However, their relationship with research measures when assessing individuals at high risk of addiction and cardiovascular disease is not well understood.

We have previously reported the presence of several cardiovascular risk factors including obesity and alcohol abuse and dependence in a community sample of American Indians and Mexican Americans residing in San Diego County (e.g., [4, 26–29]). There is also evidence to suggest a relationship between these cardiovascular risk factors and reduced cardiovagal control ([30–38]; but see [39]). However, the relationship between cardiac autonomic control and cardiovascular risk factors in these two populations is not well understood. The objective of the present study was to determine associations among obesity, hypertension, and cardiovascular autonomic control in a selected sample of American Indian and Mexican American men and women residing in the same geographical region. This study also assessed the effects of gender in these associations.

## 2. Methods

### 2.1. American Indian and Mexican American Participants

American Indians (*n* = 49) who were of at least one-sixteenth American Indian heritage were targeted for study participation and were recruited from eight geographically contiguous Indian reservations with a total population of ~3000 individuals. Participants who were mobile and without serious medical illness and between the ages of 18 and 72 years were recruited by using a combination of a venue-based method for sampling hard-to-reach populations and a respondent-driven procedure, as reported previously [40]. The protocol for the study was approved by two institutional internal review boards and the Indian Health Council, a tribal review group overseeing health issues for the reservations where recruitments took place.

Mexican American participants (*n* = 83) were recruited using a commercial mailing list that provided the addresses of individuals with Hispanic surnames in 11 zip codes in San Diego County within 25 miles of the research site that had a population of at least 20% Hispanic heritage residents. The mailed invitation stated that potential participants must be of Mexican American heritage, must be between the ages of 18 and 38 years, must be residing in the United States legally, and must be able to read and write in English. Potential participants were requested to phone research staff for more information. During the phone interview, potential participants were screened for the presence of the inclusion criteria listed on the invitation. Participants from both ethnic groups were excluded if they were pregnant or nursing, currently had a major medical or neurologic disorder, or had a head injury. All participants were asked to refrain from alcohol or any other substance use for 24 hours before testing. Participants were compensated for their time spent in the study.

### 2.2. Psychiatric Diagnoses

On the test day, after a complete description of the study to the participants, written informed consent was obtained using a protocol approved by the Institutional Review Board of The Scripps Research Institute. During the screening period, the study coordinator noted whether the participant was agitated, tremulous, or diaphoretic. Participants also took an alcohol breathalyzer test to assess blood alcohol concentration. Participants were eliminated from the current data analyses if they were taking psychoactive medication or had a positive breath alcohol test on the day of the evaluation. Information on demography, personal medical and psychiatry history, drinking history, and family history of alcohol and other substance dependence was obtained using a family history interview and the face-to-face Semistructured Assessment for the Genetics of Alcoholism (SSAGA). Studies that have evaluated the concurrent diagnostic validity of the SSAGA have shown that it is a highly reliable and valid instrument for use in studies of psychiatric disorders, including substance dependence [41, 42]. Diagnoses of lifetime alcohol dependence and nicotine dependence were made on the basis of DSM-IV criteria (without clustering) generated by the SSAGA. The SSAGA interviews were administered by trained research assistants, and all best final diagnoses were made by a research psychiatrist/addiction specialist (DAG). Current smoking and alcohol consumption were defined as at least one cigarette or one alcohol drink during the past two weeks.

### 2.3. Physiological Analysis and Acquisition

#### 2.3.1. Electrocardiographic, Respiration, and Blood Pressure (BP) Measures

Respiration rate and the electrocardiogram (EKG) were measured using a physiological monitoring system (Nexus-10 and BioTrace software, Mind Media, The Netherlands; or, the I-330-C2+ 12 interface and Physiolab (Research Mode) software, J&J Engineering, Poulsbo, WA, USA). The EKG was recorded using a three-lead cardiac monitoring system at a sampling rate of 1024 Hz. Systolic and diastolic BPs were measured with a Digital Blood Pressure Monitor (HEM-907XL, Omron Healthcare, Bannock, IL, USA).

#### 2.3.2. General Physiological Procedures

Participants were seated in a private room and indices of cardiovascular autonomic control were assessed by recording the EKG and the rate of respiration. During the first part of the assessment, continuous recording of EKG and rate of respiration were performed during a 5 min rest. Overall, HRV (SDNN), RSA, RMSSD, pNN50, CSI, HF-HRV, and LF-HRV were determined during this 5 min period. During the second part of the assessment, the HR response to deep breathing (HR_DB_) was assessed. Participants were asked to breathe 8 times at 6 breaths per minute using the Pacer EZ-Air Plus (Biofeedback Foundation of Europe, London, UK). The HR_DB_ was obtained by determining the mean HR range (maximum–minimum) of the five consecutive largest responses, as described by Low and Sletten [24]. In addition, mean systolic and diastolic BPs were determined throughout this recording session at 4 time points: (1) when the participant arrived to the room, (2) before and (3) after placement of EKG electrodes and respiration belt, and (4) after completion of the 5 min recording session. Procedures were performed between 9:00 AM and 2:00 PM.

### 2.4. Data Collection and Statistical Analysis

The present study assessed the relationship between obesity, cardiovascular autonomic control, and systolic and diastolic mean BPs in American Indian and Mexican American men and women at high risk of addiction and cardiovascular disease (American Indians, *n* = 49; Mexican Americans, *n* = 83; total sample = 132). Overall, HRV was assessed by calculating the standard deviation of the normal-to-normal interbeat intervals (SDNN) [43, 44]. Cardiovascular vagal control was assessed by estimating respiratory sinus arrhythmia (RSA), RMSSD (the square root of the mean squared successive differences between IBIs), pNN50 (the percentage of the absolute differences between consecutive IBIs that are greater than 50 ms), HF-HRV (high-frequency HRV), and the HR response to deep breathing (HR_DB_) [11, 18–21, 24, 25, 44]. Cardiac sympathetic control was assessed by estimating the cardiac sympathetic index (CSI) [23]. Assessment of the LF-HRV (low-frequency HRV) was performed to estimate the combined contributions of the SNS and PNS to HR [45–47]. Body mass index (BMI) was calculated as body weight in pounds divided by height in inches squared ×703 (lb/in^2^ × 703). Obesity was defined as BMI ≥ 30. It is recommended that respiration rate of participants to be within the frequency band used to define cardiovagal control (e.g., 0.12–0.40 Hz) [11, 21, 44]. The rate of respiration was measured when SDNN, CSI, RSA, RMSSD, pNN50, HF-HRV, and LF-HRV were assessed during the 5 min rest.

The EKG was manually inspected for artifacts and ectopic heart beats and filtered using the Kubios HRV Analysis Software (Biosignal Analysis and Medical Imaging Group (BSAMIG), Department of Physics, University of Kuopio, Finland; http://kubios.uef.fi/). Fourier transform was performed on the IBI data to assess HF-HRV (0.12–0.40 Hz) and LF-HRV (0.04–0.12 Hz) using the Kubios HRV Analysis Software. R-R intervals from the QRS complex of the EKG were used to generate interbeat interval (IBI) series and to estimate the SDNN, CSI, RMSSD, and pNN50 using the CMetX software, as previously described [11, 48]. To estimate RSA, the IBI series was converted into a time series sampled at 10 Hz and filtered using an optimal finite impulse response digital filter (0.12–0.40 Hz) [11]. RSA estimation was determined as the natural log of the variance of the filtered waveform [11]. HR_DB_ was estimated as described above and by Low and Sletten [24]. Hypertension was defined as mean systolic BP of at least 140 mm Hg or mean diastolic BP of at least 90 mm Hg [49]. Prehypertension was defined as systolic BP between 120 and 139 mm Hg or diastolic BP between 80 and 89 mm Hg [50].

Comparisons between demographics, obesity, lifetime history of alcohol and nicotine dependence, current smoking, and alcohol use were conducted using ANOVA for continuous variables and Fisher's exact test for dichotomous variables. Two-way ANOVA was used to determine the effects of obesity and gender on cardiovascular (HR and systolic and diastolic BPs) and autonomic (SDNN, CSI, RSA, RMSSD, pNN50, HF-HRV, LF-HRV, and HR_DB_) measures. Participants' ages were included as a covariate. Pearson's correlation was used to assess the relationship among HR and cardiovascular autonomic measures. Obesity (nonobese versus obese) and Gender (men versus women) were assessed as between subject factors. When appropriate, post hoc analysis of the two-way ANOVA utilized independent one-way ANOVAs to assess group differences. Statistical significance was set at probability level of *P* < 0.05. Power analyses indicated that there was sufficient power (.80) at *α* = 0.05 to detect differences in our primary analyses, for a medium effect size [51].

## 3. Results

### 3.1. Descriptive Data

Demographic data on the 132 participants are presented in Table 1. The sample contained more women participants (*n* = 75, 57%) than men (*n* = 57, 43%). Thirty percent (*n* = 39) of participants had a lifetime diagnosis of alcohol dependence and 48% of participants (*n* = 63) reported alcohol use sometime during the previous two weeks before the assessment. Participants currently using alcohol showed no differences in BMI, compared to controls (data not shown). However, participants with a lifetime diagnosis of alcohol dependence had greater BMI levels than their control group (alcohol dependence: 31.9 ± 1.1; no alcohol dependence: 29.2 ± 0.7; *F* (1,128) = 4.4, *P* < 0.05).

Seventeen percent (*n* = 23) of participants had a lifetime history of nicotine dependence and 22% of participants (*n* = 29) reported tobacco use sometime during the previous two weeks before the assessment. No significant changes in BMI levels were found in participants with a lifetime diagnosis of nicotine dependence or current tobacco use (data not shown). However, participants with systolic and diastolic hypertension were more likely to be obese (see Table 1).

### 3.2. Cardiovascular Responses in American Indian and Mexican American Participants: Relation to Obesity and Gender

**Figure 1:**
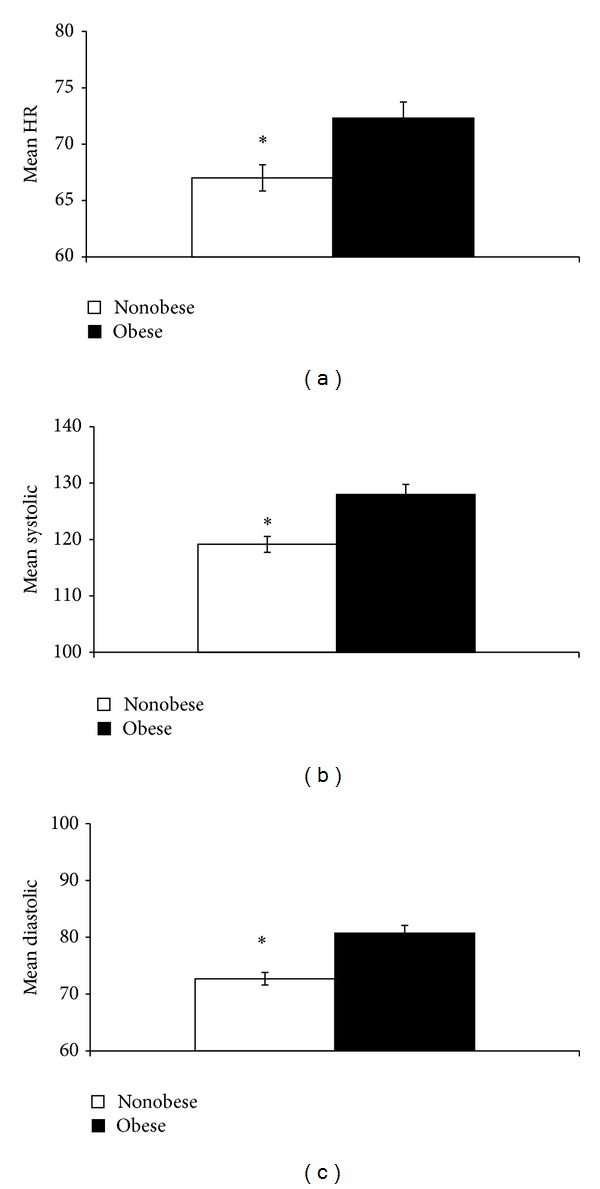
Obese participants showed a significant increase in HR (a), systolic BP (b), and diastolic BP (c) than nonobese participants. *P < 0.05.

**Figure 2:**
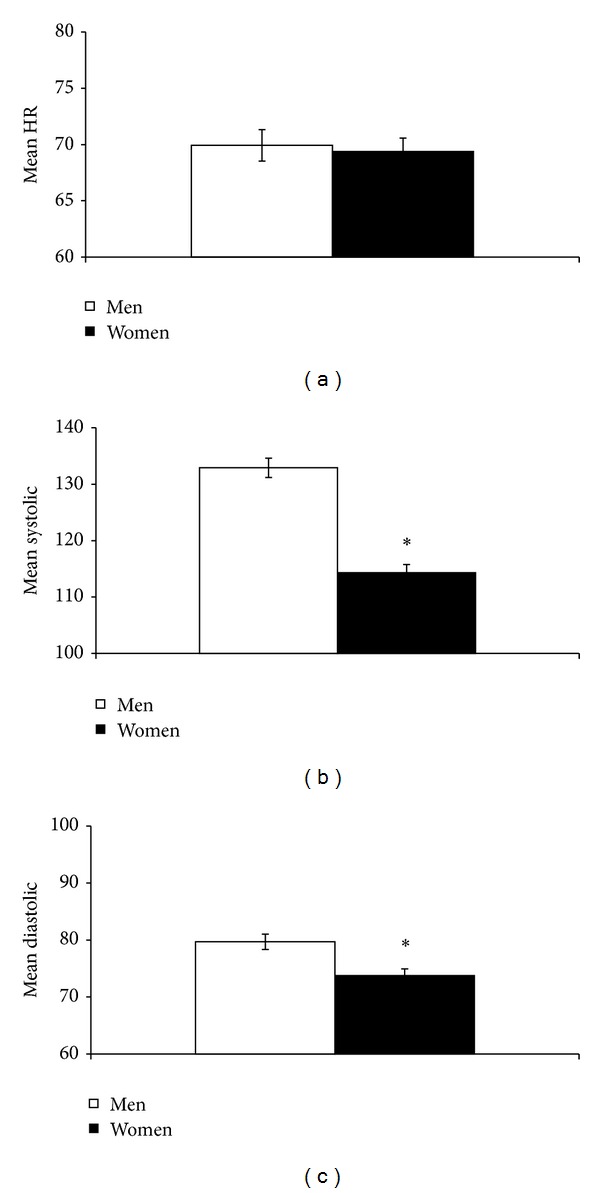
Men showed a significantly greater systolic BP (b) and diastolic BP (c), but not HR, than women. *P < 0.05.

Two-way ANOVA using age as a covariate revealed a significant obesity × gender interaction in mean HR responses (*F* (1,126) = 5.8, *P* < 0.05). Post hoc comparisons showed that obese men exhibited a significantly greater mean HR than nonobese men (obese men: 74.6 ± 3.1; nonobese men: 65.2 ± 1.6; *F* (1,54) = 8.6, *P* < 0.01). Two-way ANOVA also revealed a significant main effect of obesity in mean HR (*F* (1,126) = 8.0, *P* < 0.01; Figure 1(a)). In contrast, no significant main effect of gender was found in mean HR (Figure 2(a)).

In contrast, two-way ANOVA did not show a significant obesity × gender interaction in mean systolic and mean diastolic BPs. However, significant main effects of obesity (*F* (1,125) = 15.2, *P* < 0.001) and gender (*F* (1,125) = 69.1, *P* < 0.001) were found in mean systolic BP. Obese participants showed a significantly greater mean systolic BP than nonobese participants (Figure 1(b)). Men also showed significantly greater mean systolic BP than women (Figure 2(b)).

Significant main effects of obesity (*F* (1,125) = 19.3, *P* < 0.001) and gender (*F* (1,125) = 11.0, *P* < 0.005) were also found in mean diastolic BP. Obese participants showed a significantly greater mean diastolic BP than nonobese participants (Figure 1(c)), whereas men showed significantly greater mean diastolic BP than women (Figure 2(c)).

Gender differences were also found in the development of systolic prehypertension (120–139 mm Hg) and hypertension (at least 140 mm Hg). Participants with systolic prehypertension and hypertension were more likely to be men (Table 2). Fifty-nine percent of men (33/56) and 28% of women (21/74) exhibited systolic prehypertension. Consistent with these findings, 23% of men (13/56) and 5% of women (4/74) exhibited systolic hypertension. In contrast, diastolic prehypertension (80–89 mm Hg) and hypertension (at least 90 mm Hg) did not show gender differences (Table 2).

### 3.3. Cardiovascular Autonomic Responses in American Indian and Mexican American Participants: Relation to Obesity

Intercorrelations of metrics measuring mean HR, PNS, and SNS are shown in Table 3. Mean HR showed a positive correlation with CSI and a negative correlation with SDNN, RSA, RMSSD, pNN50, HF-HRV, and LF-HRV. Mean HR did not show a significant correlation with HR_DB_. Metrics associated with cardiovagal control (RSA, RMSSD, pNN50, HR-HRV, and HR_DB_) were positively correlated. The putative cardiac sympathetic metric, CSI, significantly negatively correlated with RSA, RMSSD, pNN50, HF-HRV, and SDNN, but not with HR_DB_. LF-HRV, suggested to assess a combination of cardiosympathetic and cardiovagal controls, significantly positively correlated with SDNN, RSA, RMSSD, pNN50, HF-HRV, and HR_DB_. LF-HRV did not show a significant correlation with CSI.

Two-way ANOVA did not show a significant obesity × gender interaction in SDNN and HR_DB_. Significant main effects of obesity were found in HR_DB_ (*F* (1,126) = 7.8, *P* < 0.01), but not in SDNN (*F* (1,126) = 2.2, *P* > 0.05). Obese participants showed a significantly reduced HR_DB_ than nonobese participants (obese men: 20.1 ± 1.0; nonobese men: 23.8 ± 0.8).

**Figure 3:**
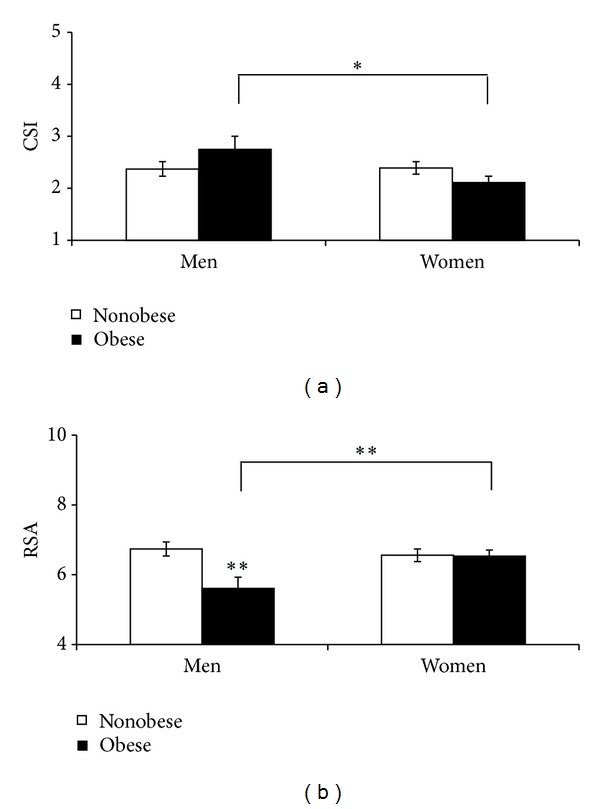
Obese men exhibited a significantly greater cardiac sympathetic index (CSI) than obese women (a). Obese men exhibited a significantly reduced cardiovagal control (RSA) than nonobese men and obese women (b). *P < 0.05; **P < 0.01.

A significant obesity × gender interaction was found in CSI (*F* (1,126) = 4.7, *P* < 0.05). Post hoc comparisons showed that obese men exhibited a significantly greater CSI than obese women (Figure 3(a), *P* < 0.05). Significant main effects of gender were found in CSI (*F* (1,126) = 4.4, *P* < 0.05). Men showed significantly greater CSI than women (women: 2.25 ± 0.097; men: 2.56 ± 0.12, *P* < 0.05). Two-way ANOVA also revealed significant obesity × gender interaction in RSA (*F* (1,126) = 6.9, *P* < 0.05). Post hoc comparisons showed that obese men exhibited a significantly reduced RSA than nonobese men (Figure 3(b), *P* < 0.005). Post hoc assessment showed that obese men exhibited a significantly reduced RSA than obese women (Figure 3(b), *P* < 0.01). In contrast, other indices of cardiovagal control showed different results. Two-way ANOVA did not show a significant obesity × gender interaction in RMSSD, pNN50, and HF-HRV (*F*'s (1,126) < 2.9, *P* > 0.05). While no significant main effects of obesity were found in indices of cardiovagal control (*F*'s (1,126) < 1.3, *P* > 0.05); RSA showed a nonsignificant trend toward reduction in obese individuals (obese: 6.2 ± 0.2; nonobese: 6.6 ± 0.1; *F* (1,126) = 4.8, *P* = 0.05). No significant obesity × gender interaction or main effect of Obesity was found in LF-HRV. 

Two-way ANOVA revealed no significant obesity × gender interaction in respiration rates (*F* (1,99) = 0.037, *P* > 0.05). Obese and nonobese participants showed similar respiration rates (*F* (1,99) = 2.6, *P* > 0.05; obese: 15.5 ± 0.5 breaths/min; nonobese: 14.5 ± 0.4 breaths/min). Respiration rates were also not different between men and women (*F* (1,99) = 3.4, *P* > 0.05; men: 14.4 ± 0.5 breaths/min, women: 15.6 ± 0.4 breaths/min). These respiration rates were within the frequency band used to define RSA and HF-HRV (e.g., 0.12–0.40 Hz or 7 to 24 breaths/min).

## 4. Discussion

The prevalence of obesity and increased risk of cardiovascular diseases have been reported in both American Indians and Mexican Americans [1–3, 5, 8]. Studies suggest that when compared to other race/ethnic groups, American Indians have one of the highest rates of cardiovascular disease and the highest prevalence of stroke in noninstitutionalized adults [52–54]. Mexican Americans have also shown a higher incidence of stroke than non-Hispanic whites [55]. The present study assessed the relationship among obesity, cardiovascular autonomic control, and systolic and diastolic BPs in a community sample of American Indian and Mexican American men and women residing in San Diego County. Results from this study are consistent with the strong evidence from epidemiological and physiological studies showing a relationship between obesity and hypertension (see [56, 57]). Findings from the present study also showed that men had significantly greater systolic and diastolic BPs and were more likely to develop systolic prehypertension and hypertension than women. Obese men showed lower RSA, an index of cardiovagal control, than nonobese men. Obese men also exhibited lower cardiovagal control and greater cardiac sympathetic activity than obese women. These findings suggest gender differences in cardiovascular autonomic control that may contribute to the greater incidence of systolic prehypertension and hypertension in American Indian and Mexican American men, compared to women.

Intercorrelations of metrics measuring cardiovagal control (RSA, RMSSD, pNN50, and HF-HRV) showed a significant correlation with HR_DB_. These findings suggest a close relationship among these experimental and clinical measures of cardiovagal control. The estimate of RSA used in the present study had been shown to significantly positively correlate with other metrics of cardiovagal control as well as the time-series RSA index developed by Porges and colleagues [11, 58]. SDNN, a measure of overall HRV, positively correlated with all metrics of cardiovagal control and negatively with mean HR and the putative sympathetic metric CSI. Consistent with these findings, CSI significantly negatively correlated with all experimental measures of cardiovagal control (RSA, RMSSD, pNN50, and HF-HRV). These negative correlations between CSI and cardiovagal time-domain measures are consistent with studies in participants assessed at rest by Allen and colleagues [11]. In contrast, CSI did not correlate with HR_DB_. These findings were expected since HR_DB_ was not assessed at rest, but during paced breathing set at 6 bpm. Evidence suggests that LF-HRV indexes a combination of cardiosympathetic and cardiovagal controls [20, 21]. 

Results from this study showed that LF-HRV significantly positively correlated with all measures of cardiovagal control (RSA, RMSSD, pNN50, HF-HRV, and HR_DB_), and overall HRV (SDNN), negatively correlated with mean HR and did not correlate with CSI. These findings suggest that cardiovagal control appears to play a more significant influence over the LF-HRV power (0.04–0.12 Hz) than cardiosympathetic control in the sample assessed in the present study. These results are also consistent with previous studies showing that LF-HRV power significantly positively correlated with baroreflex-cardiovagal function, but not with cardiac sympathetic control [45, 47, 59]. Studies with a larger sample of participants are needed to assess the relationship between metrics of cardiovascular autonomic control and measures of stroke risk that are independent of mean arterial pressure such as blood pressure variability [60].

A significant obesity × gender interaction was found in CSI and RSA. Subsequent post hoc analyses showed that obese men exhibited lower RSA and higher CSI than obese women. These data suggest gender-related differences in autonomic cardiovascular control in obese individuals. Gender differences in the overall sample were also observed in cardiac sympathetic activity with men showing greater CSI than women. Moreover, gender differences were found in the incidence of systolic prehypertension and hypertension in this sample of American Indian and Mexican American men, compared to women. While statistical analyses did not find obesity × gender interaction in mean systolic BP, systolic hypertension was more frequently found in obese participants. Further studies are needed to determine whether activation of the SNS plays a role in the increased incidence of prehypertension and hypertension in men, compared to women. A recent meta-analysis found that individuals within the high-pre-hypertensive range have a significant risk of future stroke [61]. There is evidence to suggest that stroke, among several vascular diseases, is the condition that most highly correlates with BP [62]. Therefore, the greater incidence of prehypertension in men than in women suggests that this sample of American Indian and Mexican American men may have a greater risk for stroke than women.

In contrast, two-way ANOVA using age as a covariate showed that other metrics of cardiovagal control (RMSSD, pNN50, and HF-HRV) or the combined cardiosympathetic-cardiovagal measure control (LF-HRV) were not significantly different between obese and nonobese groups. The present study also found that while RSA was significantly reduced in obese men, CSI was not significantly different between groups. These findings suggest that reduced PNS cardiovascular control may have a stronger association with obesity than an increase in SNS cardiovascular control in this sample of American Indians and Mexican Americans. However, the cardioautonomic and cardiovascular mechanisms mediating these findings are not well understood. While the present study and previous reports have shown a significant positive correlation among experimental metrics of cardiovagal control (e.g., [11]), differences in their sensitivity to assess PNS cardiovascular control need to be addressed. Future studies are also needed to assess the relative contributions of baroreflex-cardiovagal function to these findings. 

Findings from the present study showed that mean HR values were not different between men and women. Previous studies using 24 hr EKG recordings have demonstrated that women exhibited faster mean HR than men (e.g., [63, 64]). While our findings are in contrast with these previous reports, differences in the sample distribution may have contributed to these differences. For instance, Umetani and colleagues [64] found that gender-related differences in mean HR were observed in young-adults, but not in middle-aged individuals. However, the percent of participants that were middle-aged (40–65 years of age) was not different between men and women (men: 7% (*n* = 4/57) versus women: 8% (*n* = 6/75)) in the sample studied. Methodological differences may have also contributed to differences between studies. Ramaekers et al. [63] and Umetani et al. [64] used 24 hr EKG recordings, whereas the present study used 5 min recordings. Consistent with this observation, an earlier study by Ryan and colleagues [65] using 8 min EKG recordings showed higher mean HR in men than in women (20 to 39 years of age). These findings suggest that gender-related differences in mean HR may be dependent on the length of the recording period. In the present study, shorter recording times (5 min) were used to assess mean HR and measures of cardiovascular autonomic control eliminating circadian contributions to these dependent variables (see [44]).

American Indians and Mexican Americans have an increase in some risk factors for cardiovascular disease such as alcohol and tobacco use [9]. We have previously reported the presence of several cardiovascular risk factors including alcohol abuse and dependence in this sample of American Indians and Mexican Americans (e.g., [4, 26–29]). However, our current results show that the incidence of alcohol and tobacco use and dependence is not different between obese and nonobese participants. These data suggest that these risk factors for cardiovascular disease do not explain the differences between obese and nonobese groups. While rates of current alcohol use and lifetime diagnosis of alcohol dependence were not different between obese and nonobese participants, participants with a lifetime diagnosis of alcohol dependence had greater BMI levels than those without a history of alcohol dependence. We have previously reported a greater incidence of alcohol dependence in men than in women in American Indians (men: 65%; women: 54%) and Mexican Americans (men: 33%; women: 23%) in this sample population [29, 66]. Since there is evidence of a relationship between alcohol dependence and reduced cardiovagal control [31, 33, 35–37], gender differences in alcohol dependence may also result in deficits in cardiovagal control that are more prevalent in men. Further research is needed with a larger sample to assess the role of alcohol dependence on the relationship between obesity, cardiovascular autonomic control, and hypertension in this group of American Indians and Mexican Americans.

The results of this study should be interpreted in the context of several limitations. First, while previous studies have shown the validity of CSI as an index of sympathetic activity [11, 23, 67], the data is limited and replication of pharmacological studies needs to be conducted. Nevertheless, this putative cardiac sympathetic metric significantly negatively correlated with all experimental measures of cardiovagal control and overall HRV. Second, only retrospective and cross-sectional data on the lifetime history of alcohol dependence were assessed. Some participants may have had symptoms of alcohol dependence at different times before the autonomic assessment. Third, the study focused on American Indian and Mexican American adults legally residing in US and it may not be possible to generalize these results to other American Indians, all Mexican Americans or all Hispanic Americans. Despite these limitations, this report represents an important step in an ongoing investigation to determine genetic and environmental risk factors associated with substance use disorders and related psychiatric disorders in these high risk and understudied ethnic groups. Separate studies in these populations with a larger sample are also needed to assess the role of cardiovascular autonomic control in the development of comorbid cardiovascular and neuropsychiatric disorders.

## 5. Conclusions

These results suggest that obesity and gender differences in cardiovascular autonomic control may contribute to risk for cardiovascular disorders in this sample of American Indians and Mexican Americans.

## Figures and Tables

**Table 1:** Demographic characteristics of participants as function of obesity.

Demographic	Total sample	Nonobese	Obese
Variables	(*n* = 132)	(*n* = 77)	(*n* = 55)
Age, in years, mean (SEM)	28.1 (0.9)	26.4 (1.1)	30.3 (1.4)*
Men	27.7 (1.2)	26.0 (1.7)	30.5 (2.1)
Women	28.3 (1.3)	26.8 (1.5)	30.2 (1.7)
Gender, *n*			
Men	57	35	22
Women	75	42	33
Years of education (SEM)	12.8 (0.2)	13.0 (0.2)	12.6 (0.2)
Current alcohol use, *n*			
No	69	35	34
Yes	63	42	21
Lifetime history of alcohol dependence, *n*			
No	92	58	34
Yes	39	18	21
Current smoking, *n*			
No	103	60	43
Yes	29	17	12
Lifetime history of nicotine dependence, *n*			
No	109	64	45
Yes	23	13	10
Systolic prehypertension, *n*			
No	76	46	30
Yes	54	30	24
Systolic hypertension, n			
No	113	70	43*
Yes	17	6	11
Diastolic pre-hypertension, *n*			
No	110	64	46
Yes	20	12	8
Diastolic hypertension, *n*			
No	113	73	40**
Yes	17	3	14

Notes: The obese group versus the nonobese group was compared using Fisher's exact test for dichotomous variables and analysis of variance (ANOVA) for continuous variables (*P < 0.05; **P < 0.001). Values are $\overset{-}{x}$  ± SEM.

**Table 2:** Effects of gender on prehypertension and hypertension.

Variable	Total sample	Men	Women
	(*n* = 130)	(*n* = 56)	(*n* = 74)
Systolic prehypertension, *n*			
No	76	23	53*
Yes	54	33	21
Diastolic prehypertension, *n*			
No	110	49	61
Yes	20	7	13
Systolic hypertension, *n*			
No	113	43	70*
Yes	17	13	4
Diastolic hypertension, *n*			
No	113	45	68
Yes	17	11	6

Notes: Groups were compared using Fisher's exact test for dichotomous variables (*P < 0.005).

**Table 3:** Intercorrelations between metrics of mean HR, overall HRV, cardiovagal control, and cardiac sympathetic activity.

	Mean HR	SDNN	CSI	RSA	RMSSD	pNN50	HF-HRV	LF-HRV	HR_DB_
Mean HR	—								
SDNN	−0.465**	—							
CSI	0.543**	−0.266*	—						
RSA	−0.519**	0.864**	−0.542**	—					
RMSSD	−0.590**	0.867**	−0.609**	0.841**	—				
pNN50	−0.662**	0.788**	−0.670**	0.857**	0.922**	—			
HF-HRV	−0.252*	0.822**	−0.344**	0.737**	0.815**	0.679**	—		
LF-HRV	−0.206*	0.677**	0.028	0.467**	0.450**	0.356**	0.428**	—	
HR_DB_	0.075	0.494**	−0.158	0.553**	0.413**	0.407**	0.433**	0.344**	—

Note: Mean HR: mean heart rate; SDNN: standard deviation of interbeat interval (IBI); CSI: Toichi cardiac sympathetic index (sympathetic-related variability); RSA: natural log of variance of filtered (0.12–0.40 Hz) IBI; RMSSD: root mean square of successive differences between IBIs; pNN50: the percentage of the absolute differences between consecutive IBIs that are greater than 50 ms; HF-HRV: high frequency (HF)-heart rate variability (HRV) power (0.12–0.40 Hz); LF-HRV: low frequency (LF)-HRV power (0.04–0.12 Hz); HR_DB_: the HR response to deep breathing at 6 breaths per minute. *Indicates that Pearson's correlation is significant at *P* < 0.05. **Indicates that Pearson's correlation is significant at *P* < 0.001.
